# Serological Evidence for Non-Lethal Exposures of Mongolian Wild Birds to Highly Pathogenic Avian Influenza H5N1 Virus

**DOI:** 10.1371/journal.pone.0113569

**Published:** 2014-12-15

**Authors:** Martin Gilbert, Björn F. Koel, Theo M. Bestebroer, Nicola S. Lewis, Derek J. Smith, Ron A. M. Fouchier

**Affiliations:** 1 Wildlife Conservation Society, 2300 Southern Blvd, Bronx, NY, 10460, United States of America; 2 Boyd Orr Centre for Population and Ecosystem Health, College of Medical, Veterinary and Life Sciences, University of Glasgow, Glasgow, G12 8QQ, United Kingdom; 3 Department of Viroscience, Erasmus Medical Center, 3015GE, Rotterdam, The Netherlands; 4 Department of Zoology, University of Cambridge, Cambridge, CB2 3EJ, United Kingdom; Rutgers University, United States of America

## Abstract

Surveillance for highly pathogenic avian influenza viruses (HPAIV) in wild birds is logistically demanding due to the very low rates of virus detection. Serological approaches may be more cost effective as they require smaller sample sizes to identify exposed populations. We hypothesized that antigenic differences between classical Eurasian H5 subtype viruses (which have low pathogenicity in chickens) and H5N1 viruses of the Goose/Guangdong/96 H5 lineage (which are HPAIV) may be used to differentiate populations where HPAIVs have been circulating, from those where they have not. To test this we performed hemagglutination inhibition assays to compare the reactivity of serum samples from wild birds in Mongolia (where HPAIV has been circulating, n = 1,832) and Europe (where HPAIV has been rare or absent, n = 497) to a panel of reference viruses including classical Eurasian H5 (of low pathogenicity), and five HPAIV H5N1 antigens of the Asian lineage A/Goose/Guangdong/1/96. Antibody titres were detected against at least one of the test antigens for 182 Mongolian serum samples (total seroprevalence of 0.10, n = 1,832, 95% adjusted Wald confidence limits of 0.09–0.11) and 25 of the European sera tested (total seroprevalence of 0.05, n = 497, 95% adjusted Wald confidence limits of 0.03–0.07). A bias in antibody titres to HPAIV antigens was found in the Mongolian sample set (22/182) that was absent in the European sera (0/25). Although the interpretation of serological data from wild birds is complicated by the possibility of exposure to multiple strains, and variability in the timing of exposure, these findings suggest that a proportion of the Mongolian population had survived exposure to HPAIV, and that serological assays may enhance the targeting of traditional HPAIV surveillance toward populations where isolation of HPAIV is more likely.

## Introduction

Since its emergence in 1997, a highly pathogenic strain of avian influenza virus (HPAIV) subtype H5N1 has affected 64 countries and is now enzootic in parts of Asia and Africa [1]. Outbreaks have led to heavy losses of domestic poultry, and although absolute numbers of human infections remain relatively small, concerns persist that minor genetic mutations could produce a pandemic virus [2], [3]. While the impact of HPAIV H5N1 has been greatest within the domestic poultry sector, the role of wild birds in viral persistence and spread remains unresolved [4]. Much of our understanding arises from studies of birds that are clinically affected or dead [5]–[7], but attempts to study the virus in the more epidemiologically relevant live birds has proven challenging.

Detection of HPAIV antigen in live wild birds is logistically demanding. Given the transient nature of influenza virus infections (with less than ten days of viral shedding [8], [9]), very large sample sizes are required to attain acceptable levels of detection probability [10]. This is further compounded by variation in species susceptibility to HPAIV infection [8], and potential for spatial and temporal fluctuations in prevalence [10], [11]. Successful surveillance for HPAIV in wild bird populations therefore requires that efforts be directed at the correct species at the correct place and time, and be of sufficient scale to detect circulating virus. For these reasons it is perhaps unsurprising that antigen-directed surveillance techniques have been largely unsuccessful in detecting HPAIV in free-ranging wild birds.

Serological approaches to HPAIV surveillance may represent a more cost effective strategy, but must overcome a number of challenges. Antibodies to the surface glycoprotein hemagglutinin (HA) can be measured using several techniques (such as hemagglutinin inhibition [HI], and microneutralisation assays). These antibodies persist for prolonged periods enabling assessment of seroprevalence based on a relatively modest sample size [12]. Therefore, while the per-sample cost of serological surveillance may be high, the potential to draw conclusions from a smaller sample sizes could reduce overall costs of surveillance programs. However, individual tests cannot differentiate between HA antibodies arising from infections with high versus low pathogenic avian influenza viruses (LPAIV) and remains an important obstacle to their use in HPAIV surveillance.

Unlike most influenza viruses in wild birds, highly pathogenic strains of the H5 subtype are genetically highly variable [13], due to accumulated mutations in the HA gene and corresponding amino acid substitutions in the HA protein that give rise to the phenomenon called ‘antigenic drift’ [14]. Assays such as HI that measure the reactivity between antibodies and antigens have shown that genetically distinct viruses may also differ antigenically [15], [16]. The objective of this study is to test the hypothesis that low pathogenic classical Eurasian H5 viruses (CE-LP-H5) and highly pathogenic H5 viruses from the Asian lineage A/Goose/Guangdong/1/96 (GG-HP-H5) differ antigenically, and that this may be used to differentiate wild bird populations where HPAIVs have been circulating (e.g. Mongolia, **S1** and **S2 Figures**), from those where they have not (e.g. Europe, **S1** and **S3 Figures**). To test this we used HI assays and titre comparison to assess the viability of serosurveillance as a means of identifying wild bird populations that have been exposed to HPAIV.

## Materials and Methods

### Sample selection

Comparative sets of serum samples were selected to represent geographic regions with a history of HPAIV outbreaks in wild birds (Mongolia), and areas where no wild bird outbreaks of HPAIV had been recorded (Norway and The Netherlands, referred collectively as Europe). As far as possible sample sets were matched taxonomically, with a bias toward species within the order Anseriformes that are believed to represent the principal reservoir of all influenza viruses [17].

A total of eight HPAIV H5N1 outbreaks have been reported in Mongolia between 2005 and 2013 [11]. Using the World Health Organization classification system for the nomenclature of HPAIV H5N1 [18], these outbreaks included four involving viruses from clade 2.2 at two sites in the aimags (provinces) of Bulgan and Khovsgol during 2005 and 2006, and clade 2.3.2.1 at four sites in Arkhangai, Khovsgol and Sukhbaatar aimags during 2009 and 2010 [11]. Serum samples were collected from wild waterbirds in Arkhangai, Bulgan and Khovsgol between July 2008 and August 2009, and included birds captured during HPAIV outbreaks at Erhel Nuur (July 2009), and Doroo Tsagaan Nuur (August 2009) [11].

Samples representing Europe were collected in the Netherlands and Norway (Spitsbergen) between July 2005 and February 2012 (hereafter referred to as the European sample set). There have been no cases of HPAIV H5N1 recorded in either country, and although wild bird outbreaks have occurred elsewhere in Europe since October 2005, they have been infrequent since 2006 [19].

Sera were prescreened using a commercially available bELISA (FlockCheck AI MultiS-Screen Antibody Test Kit, IDEXX Laboratories, Westbrook, Maine, USA) to identify samples with antibodies to influenza virus nucleoprotein (NP) indicating exposure to influenza A virus following the manufacturer’s instructions. Samples testing positive for influenza A antibodies were then selected for HI analysis. These samples were all collected from live birds showing no signs of disease, with the exception of three clinically ill ruddy shelducks (*Tadorna ferruginea*) from Mongolia from which HPAIV H5N1 was diagnosed [11], and one tundra swan (*Cygnus columbianus*) with bacterial septicaemia but no HPAIV, which was sampled at Erhel Nuur in Mongolia on 12 July 2009, 18 days before HPAIV was isolated from another bird at this site.

### Hemagglutinin inhibition

Test sera underwent HI assays against a panel of six standard influenza antigens using published methods [20]. Briefly, serum samples were incubated for 16 hours at 37°C with the receptor-destroying enzyme of *Vibrio cholera* to remove non-specific inhibitors of hemagglutination activity, followed by inactivation for one hour at 56°C. Two-fold serial dilutions of test sera with a starting titre of 1∶20 were prepared using phosphate-buffered saline in U-bottom 96 well microtiter plates. These were incubated at 37°C for 30 minutes with four hemagglutinating units of one virus from the standard antigen panel. Dilutions were then incubated for one hour at 4°C with a 1% solution of turkey erythrocytes. Hemagglutination patterns were then read in duplicate. The virus panel included six 6∶2 recombinant viruses based on a PR8 backbone, with the HA and NA of diverse H5 strains including CE-LP-H5, and representatives of the Asian lineage GG-HP-H5, including viruses from H5N1 clade 0, 1, 2.1, 2.2, and 2.3 (Table 1). The sequence of the HA genes was modified to remove the multi-basic cleavage site to enable study within biosafety level 2 laboratories.

**Table 1:** Recombinant virus strains.

Clade	Strain name	Abbreviation
Classic	A/mallard/Netherlands/3/99(H5N2)	NL99
Clade 0	A/HongKong/156/1997(H5N1)	HK97
Clade 1	A/Viet Nam/1194/2004(H5N1)	VN04
Clade 2.1	A/Indonesia/5/2005(H5N1)	ID05
Clade 2.2	A/Turkey/Turkey/1/2005(H5N1)	TU05
Clade 2.3	A/Anhui/1/2005(H5N1)	AN05

Original virus strains for the hemaglutinin and neuraminidase antigens used in the standard panel of recombinant influenza A viruses used in hemagglutination inhibition assays.

Negative controls (based on serum incubated without virus) were used to measure non-specific hemagglutination by each serum sample, and used as a minimum sensitivity cut-off for the assay. Serum derived from experimental exposure studies that challenged wild bird test subjects with HPAIV strains (the birds were negative for antibodies to influenza A virus at the time of exposure) were used as positive controls ([21]–[26] Table 2).

**Table 2:** Positive control sera.

Species of test subject	Exposure strain	Exposure clade	Reference
Herring gull	A/DK/AY/AVL-1/2001(H5N1)	1	(21)
Herring gull	A/DK/AY/AVL-1/2001(H5N1)	1	(21)
Common pochard	A/turkey/Turkey/1/2005(H5N1)	2.2	(22)
Tufted duck	A/turkey/Turkey/1/2005(H5N1)	2.2	(22)
Tufted duck	A/turkey/Turkey/1/2005(H5N1)	2.2	(22)
Eurasian wigeon	A/turkey/Turkey/1/2005(H5N1)	2.2	(22)
Dunlin	A/WS/MN/244/2005(H5N1)	2.2	(23)
Dunlin	A/WS/MN/244/2005(H5N1)	2.2	(23)
Dunlin	A/WS/MN/244/2005(H5N1)	2.2	(23)
Greylag goose	A/CK/Korea/IS/2006(H5N1)	2.2	(24)
Greylag goose	A/CK/Korea/IS/2006(H5N1)	2.2	(24)
Mandarin duck	A/CK/Korea/IS/2006(H5N1)	2.2	(24)
Bar-headed goose	A/WS/MN/244/2005(H5N1)	2.2	(25)
Bar-headed goose	A/RS/MN/X63/2009(H5N1)	2.3	(26)
Bar-headed goose	A/RS/MN/X63/2009(H5N1)	2.3	(26)

Details of positive control sera from wild bird test subjects that were experimentally exposed to strains of highly pathogenic avian influenza virus of the Asian lineage A/Goose/Guangdong/1/96, H5N1. Viral strain names include the acronyms: WS = whooper swan, RS = ruddy shelduck, DK = duck, CK = chicken, AY = Anyang, MN = Mongolia.

### Ethics statement

For Mongolia, bird capture and handling permits were issued by the Mongolian Ministry of Nature, the Environment and Tourism following approval of protocols by the Institute of Biology at the Mongolian Academy of Sciences, with additional approval from the University of Minnesota, Institutional Animal Care and Use Committee (Protocol 1006A84613). Samples from European wild birds were collected under permits 122-11-31, and 122-07-09 from the National Institute of Public Health and the Environment in Bilthoven, issued by Stichting DEC Consult (for the capture and collection of samples from wild birds in the Netherlands), and permit 201000149 obtained through the University of Groningen (for the capture and sampling wild birds in Norway).

## Results

### Sample selection

A total of 1,832 serum samples were available from surveys in Mongolia, of which 479 were found to be positive for antibodies to NP using a blocking ELISA (bELISA) and selected for HI analysis. A full account of the bELISA results from Mongolia have been published elsewhere [27]. A total of 497 sera were available from surveys conducted in Europe. Of these 200 were selected for HI analysis based on the presence of antibodies to influenza A virus.

### Hemagglutinin inhibition assays

Serum samples with antibodies against influenza A virus NP underwent HI analyses against a panel of six H5 influenza virus strains including CE-LP-H5, and five GG-HP-H5 representatives including viruses from clade 0, 1, 2.1, 2.2, and 2.3 (Table 1). Titres are summarized in Table 3 and are provided in full in **S1**, **S2** and **S3 Tables**. Of the 479 Mongolian serum samples tested, 182 were found to contain detectable antibody titres to one or more of the test antigens (total seroprevalence of 0.10, n = 1,832, 95% adjusted Wald confidence limits of 0.09–0.11) while HI titres were found against at least one antigen for 25 of the 200 European sera tested (total seroprevalence of 0.05, n = 497, 95% adjusted Wald confidence limits of 0.03–0.07), and 14 of 15 positive control sera. The HA antigen HK97 was found to bind most widely with test sera, with binding antibody titres found in 171/479 of the Mongolian samples and 25/25 of the European samples.

**Table 3:** Summary of hemagglutinin inhibition (HI) titres.

Sample set								Titre (Log_2_)							No HI titre recorded
	Antigen	0	1	2	3	4	5	6	7	8	9	10	11	12	
Mongolia	NL99 [Classic]	13 (7)	33 (18)	14 (8)	11 (6)	13 (7)	4 (2)	1 (1)	0 (0)	2 (1)	0 (0)	0 (0)	0 (0)	0 (0)	91 (50)
Europe		0 (0)	9 (36)	2 (8)	4 (16)	1 (4)	0 (0)	4 (16)	1 (4)	0 (0)	0 (0)	0 (0)	0 (0)	0 (0)	4 (16)
Control		0 (0)	0 (0)	0 (0)	0 (0)	1 (7)	0 (0)	0 (0)	2 (13)	1 (7)	0 (0)	0 (0)	0 (0)	0 (0)	11 (73)
Mongolia	HK97 [0]	17 (9)	60 (33)	39 (21)	21 (12)	18 (10)	10 (5)	1 (1)	2 (1)	2 (1)	0 (0)	1 (1)	0 (0)	0 (0)	11 (6)
Europe		5 (20)	7 (28)	4 (16)	2 (8)	4 (16)	2 (8)	1 (4)	0 (0)	0 (0)	0 (0)	0 (0)	0 (0)	0 (0)	0 (0)
Control		3 (20)	1 (7)	2 (13)	2 (13)	2 (13)	0 (0)	1 (7)	0 (0)	0 (0)	2 (13)	0 (0)	1 (7)	0 (0)	1 (7)
Mongolia	VN04 [1]	7 (4)	20 (11)	9 (5)	5 (3)	2 (1)	0 (0)	1 (1)	1 (1)	0 (0)	0 (0)	0 (0)	0 (0)	0 (0)	137 (75)
Europe		0 (0)	1 (4)	1 (4)	3 (12)	0 (0)	0 (0)	0 (0)	0 (0)	0 (0)	0 (0)	0 (0)	0 (0)	0 (0)	20 (80)
Control		0 (0)	1 (7)	0 (0)	0 (0)	0 (0)	0 (0)	3 (20)	0 (0)	0 (0)	0 (0)	7 (7)	0 (0)	0 (0)	10 (67)
Mongolia	ID05 [2.1]	7 (4)	20 (11)	10 (5)	8 (4)	7 (4)	2 (1)	2 (1)	0 (0)	1 (1)	0 (0)	0 (0)	0 (0)	0 (0)	125 (69)
Europe		3 (12)	1 (4)	2 (8)	0 (0)	0 (0)	0 (0)	0 (0)	0 (0)	0 (0)	0 (0)	0 (0)	0 (0)	0 (0)	19 (76)
Control		0 (0)	0 (0)	1 (7)	0 (0)	0 (0)	1 (7)	2 (13)	0 (0)	0 (0)	0 (0)	7 (7)	0 (0)	0 (0)	10 (67)
Mongolia	TU05 [2.2]	10 (5)	30 (16)	26 (14)	15 (8)	9 (5)	4 (2)	1 (1)	1 (1)	1 (1)	0 (0)	0 (0)	0 (0)	0 (0)	85 (47)
Europe		2 (8)	3 (12)	4 (16)	0 (0)	0 (0)	0 (0)	0 (0)	0 (0)	0 (0)	0 (0)	0 (0)	0 (0)	0 (0)	16 (64)
Control		0 (0)	2 (13)	2 (13)	0 (0)	1 (7)	0 (0)	2 (13)	1 (7)	1 (7)	0 (0)	0 (0)	0 (0)	1 (7)	5 (33)
Mongolia	AN05 [2.3]	3 (2)	22 (12)	17 (9)	8 (4)	4 (2)	2 (1)	2 (1)	1 (1)	0 (0)	1 (1)	0 (0)	0 (0)	0 (0)	122 (67)
Europe		0 (0)	5 (20)	1 (4)	1 (4)	1 (4)	2 (8)	0 (0)	0 (0)	0 (0)	0 (0)	0 (0)	0 (0)	0 (0)	15 (60)
Control		0 (0)	1 (7)	1 (7)	1 (7)	0 (0)	1 (7)	0 (0)	1 (7)	1 (7)	0 (0)	0 (0)	1 (7)	8 (53)	0 (0)

Includes serum sample sets from Mongolia (x182), Europe (x25), and a positive control set (x15). Samples underwent an initial log_10_ dilution, followed by serial log_2_ dilutions, with the initial 1∶10 equivalent set to 0 on the log_2_ scale. HI titres were measured for all sera against a panel of standard influenza A virus H5 antigens, including A/mallard/Netherlands/3/1999(H5N2) (NL99), A/Hong Kong/156/1997(H5N1) (HK97), A/Viet Nam/1194/2004(H5N1) VN04, A/Indonesia/5/2005(H5N1) (ID05), A/Turkey/Turkey/1/2005(H5N1) (TU05), and A/Anhui/1/2005(H5N1) (AN05). H5 clade designation is given in square brackets. Number of samples testing positive is indicated at each titre, and expressed as a percentage of each sample set tested in parentheses.

HA antibody titres in Mongolia and Europe were generally low, with only 27.3% of Mongolian and 9.0% of European samples with measurable titres higher than 1∶20. The geometric mean value (GMV) was calculated for all titres obtained for each sample. The five sera with the highest GMVs were all from Mongolia, of which three were from birds sampled at lakes where outbreaks were ongoing, and one was the tundra swan sampled at Erhel Nuur in early July 2009, 18 days before HPAIV H5N1 was detected at this site.

The Mongolian sample set included serum from three sick ruddy shelducks that were diagnosed with HPAIV H5N1 (by RT-PCR and virus isolation [11]). Antibody titres were recorded against two (HK97 and ID05) and all six of the test antigens for two of these birds (both of which were adults, MN09A-0556 and MN09A-0969 respectively), while no antibody titres were detected for the third (a hatch year bird, MN09A-0911).

The measurable antibody titre recorded for the 15 positive control sera varied widely, with only four samples found to have titres against all six antigens (of which all were ducks, and all had at least one titre of 1∶640 or greater). All of the remaining positive control sera were obtained from Charadriiformes, geese or mandarin ducks (*Aix galericulata*), and only one of these was found to have titres to more than three of the antigens (a greylag goose [*Anser anser*], Bird #43, which recorded titres against four of the antigens, and a maximum of 1∶960). Once again measurable antibodies were recorded most frequently against HK97 (14/15 samples), followed by TU05 (10/15 samples).

### Direct comparison of titres

Individual HI tables were examined to determine whether populations in Europe and Mongolia showed a bias in titres against HPAIVs or LPAIVs. Sera where titres to GG-HP-H5 antigens (clades 1, 2.1, 2.2 and 2.3) exceeded those for the CE-LP-H5 antigen were defined as being HPAIV-biased, while those that fell below were termed LPAIV-biased. Titres to HK97 (clade 0) were excluded from the analysis as these exceeded all other titres in the majority of sera tested (157/221 Mongolian, European and positive control sera with antibody titres to H5). Samples where titres to GG-HP-H5 and CE-LP-H5 antigens were identical, or differed by one log_2_ unit or less (the error inherent in the HI assay [28]) were considered to be ambiguous and lacking in bias. The results of this analysis are presented in Table 4. All of the European sera with HI titres above the threshold were considered either LPAIV-biased, or ambiguous. By contrast 22 of the Mongolian sera were considered HPAIV-biased, 7 as LPAIV-biased, and 153 as ambiguous.

**Table 4:** Bias in serum titres.

Sample set	HPAI-biased	LPAI-biased	Ambiguous
Mongolia	22	7	153
European	-	10	15
Positive control	9	-	5

Summarizing the numbers of serum samples based on the ratio of hemaglutination inhibition (HI) titres to low pathogenic avian influenza virus (LPAIV) and highly pathogenic avian influenza virus (HPAIV) (H5N1 clades 1, 2.1, 2.2 or 2.3). Sera with titres to LPAIV exceeding the highest titres against HPAIV are termed ‘LPAIV-biased’, and sera with titres to LPAIV falling below the highest titres against HPAIV antigens are termed ‘HPAIV biased’. Sera for which titres against LPAIV and HPAIV differ by one log_2_ unit or less (the error inherent in the HI assay) are termed ‘ambiguous’.

**Figure 1:**
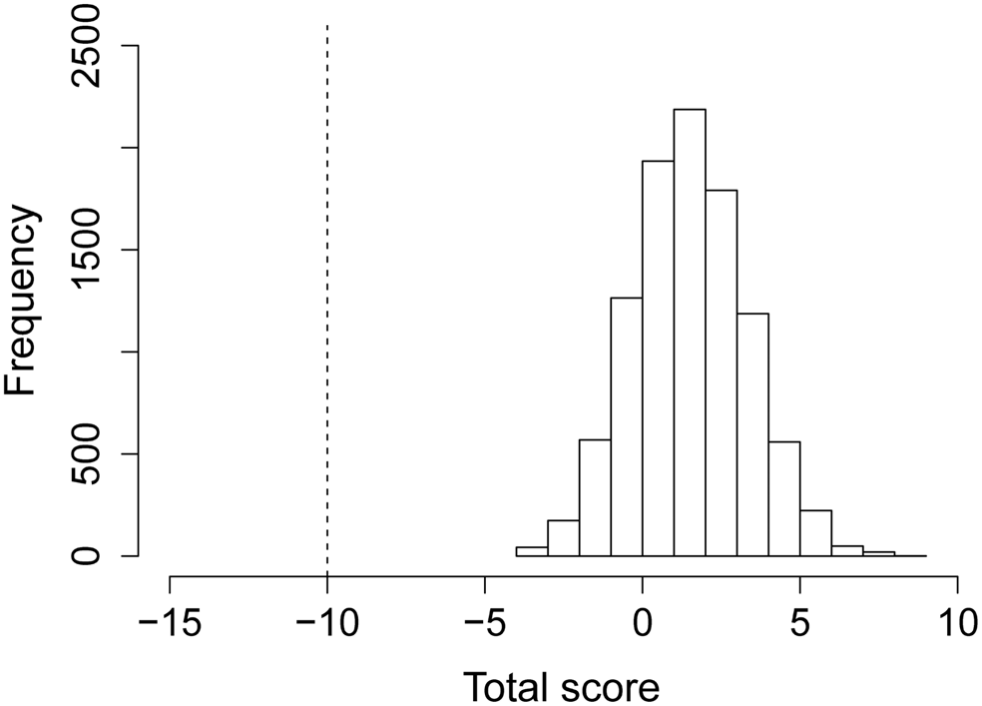
A histogram illustrating the results of a bootstrap analysis to control for sample size and determine whether European and Mongolian sample sets differed in their capacity to identify a population bias toward titres against HPAIV versus LPAIV. The analysis randomly selected 25 serum samples (equivalent to the number of European sera assessed) from the 182 Mongolian sera. Individual sera were scored +1 for HPAIV-biased sera, −1 for LPAIV-biased sera and 0 for ambiguous sera. The analysis was repeated 10,000 times and bars represent the frequency of total scores. The vertical dashed line represents the equivalent score for the European sample set.

A bootstrap analysis was performed to test whether these findings may be an artifact of the small number of European samples with antibodies to H5 (n = 25 samples). It is possible that the lack of HPAIV-biased sera in the European sample set may have resulted by chance, due to the lower number of sera with antibodies to H5. To test this, a random sample of 25 sera was selected from the Mongolian samples to determine whether the HPAIV-bias would be retained when fewer sera were considered. Samples with an HPAIV-bias were scored +1, those with an LPAIV-bias scored −1 and a score of 0 was allocated to ambiguous samples. Thus the sum of individual scores would be >0 for a population that was HPAIV-biased and <0 for an LPAIV-bias. This was repeated 10,000 times to generate a distribution of predictions (Fig. 1). The distribution of predicted scores exceeded that of the 25 European sera in all cases (as the European sample set contained more LPAIV-biased samples than the Mongolia sample set), and although 7.6% of runs predicted a population that was LPAIV-biased overall, the mean prediction was distinctly HPAIV-biased with a mean score of 2.01.

## Discussion

The theoretical basis for this study relied on a marked difference in the antigenicity of HP viruses from Classical H5 viruses, of an extent that greatly exceeded the antigenic variation present within Classical viruses. Prior experiments using ferret antisera raised against four Classical H5 viruses isolated in Europe, Asia and Africa between 1963 and 2002 found only marginal antigenic differences across a panel of 12 Classical H5 viruses isolated from domestic and wild birds between 1959 and 2002 in both Old and New World locations (S4 Table). In contrast, markedly lower titres were measured using this set of ferret antisera against viruses from the GG-HP-H5 lineage clades examined during the present study. In common with the sample set from European birds, the ferret antisera also measured high titres against the GG-HP-H5 lineage Clade 0 virus, adding support to the decision to omit this virus from the analysis due to its lack of discriminatory power.

When viewed at a population level, these results highlight several important differences in the serological profile of wild birds from Mongolia in comparison to those from Europe. Firstly, while seroprevalence data suggest that wild birds in Europe experience similar or higher levels of exposure to influenza A viruses than those in Mongolia, Mongolian birds are twice as likely to be carrying antibodies to viruses of the H5 subtype (182/1,832 in Mongolia versus 25/497 in Europe), a difference that is statistically significant (χ^2^ = 11.612, d.f. = 1, p = 0.000). When considered in isolation these results are unable to differentiate between exposures to LPAIV (represented by CE-LP-H5 virus) from those to HPAIV H5N1 (represented by GG-HP-H5 subtypes). However, by comparing relative HI titres to CE-LP-H5 and to GG-HP-H5 antigens across the populations a clear difference is evident, with a proportion of Mongolian sera found with a bias in titres against HPAIV H5N1 strains that is absent in the sample of European sera. The most likely explanation for this disparity is that at least a proportion of wild birds in Mongolia have been exposed to HPAIV H5N1and developed an antibody response to them, while those in Europe have not.

In Europe, isolation of LPAIV strains of the H5 subtype has been relatively common [29], [30] and therefore it is not unexpected that antibodies to these viruses should be found in this population. By contrast, HPAIV H5N1 has not been recorded in either of the countries where European samples were collected (the Netherlands and Norway). However, it is important to consider whether birds could have encountered the virus during their annual migrations to other countries. Samples from Spitsbergen were predominantly barnacle geese (*Branta leucopsis*), which migrate to Scotland (S3 Figure), where only a single case of HPAIV H5N1 has been recorded [19]. The Dutch samples were dominated by mallards (*Anas platyrhynchos*), represented locally by a resident and a migratory subpopulation, with migrants breeding in northwest Russia, Finland, Sweden and the Baltic states [31] (S3 Figure). Although outbreaks of HPAIV H5N1 have been recorded in several states bordering the Baltic Sea [6], [32], [33], these have been sporadic and infrequent [19]. Therefore, while it is conceivable that mallards sampled in the Netherlands could have been exposed to HPAIV H5N1 this is considered relatively unlikely.

The birds represented in the Mongolian sample are more likely to have encountered H5 viruses of high as well as low pathogenic strains. Low pathogenic strains of H5 viruses have been isolated in Mongolia in 2001, 2011 and 2013 [34], [35], and there have been eight outbreaks of HPAIV H5N1 recorded in the country since 2005 [11]. Birds may also have encountered H5 viruses during their annual movements to and from wintering areas in central and eastern China, and the Indian subcontinent including large areas where HPAIV H5N1 is enzootic (**S1** and **S2 Figures**).

On an individual bird level exposure histories are more challenging to interpret. In most cases antibody titres were relatively low, suggesting that exposures may have occurred many months or even years earlier. Anseriformes are long lived, with longevity records of 28, 40 and 24 years for wild individuals of the genera *Cygnus*, *Anser* and *Tadorna* respectively [36]. Antibody titres might therefore represent multiple exposures to H5 viruses during their lifetimes, potentially involving both low and highly pathogenic subtypes. The influence of such complex histories on the HI profile of individual birds is hard to predict. Indeed, it should be noted that both of the serum samples with H5 titres from confirmed HPAIV H5N1 cases were considered to be ambiguous with respect to their titres to LPAIV and HPAIV strains and showed no bias.

Among the Mongolian samples six species were found to have HPAIV-biased sera including bar-headed goose (*Anser indicus*, x12), bean goose (*A. fabilis*, x1), swan goose (*A. cygnoides*, x1), whooper swan (*C. cygnus*, x3), ruddy shelduck (x4) and tufted duck (*Aythya fuligula*, x1). Although HPAIV-biased sera were more commonly recorded in bar-headed geese (representing 29% of the samples found to have antibodies to H5 subtypes), this should not imply that the species has been disproportionately exposed to HPAIV H5N1 in comparison to other birds. Only 7% of bar-headed geese sampled were found to be seropositive for H5 antibodies, suggesting that repeat exposures may be less likely in this species, resulting in less complicated HI profiles. By contrast, 26% of adult ruddy shelducks have antibodies to H5 viruses, suggesting that repeat exposures may be more common, and thus individual HI profiles are more difficult to interpret.

Several positive control sera did not inhibit hemagglutination by viruses that were genetically similar to the antigens to which the birds were experimentally exposed. For instance neither of the bar-headed geese that were inoculated experimentally with Mongolia/X63/09 (a clade 2.3.2.1 virus) (Bird #81 and Bird #94) were able to inhibit hemagglutination by AN05 (a clade 2.3 virus) despite demonstrating titres against other H5 strains. This is consistent with the observation that some genetic changes may lead to disproportionately large differences in antigenicity [28]. The HA genes of Mongolia/X63/09 and AN05 are 93.0% identical at the nucleic acid level, and are 94.1% identical in their predicted amino acid sequence based on a pair-wise distance model. This is of particular importance in interpreting the HI tables for the Mongolian sera, as Mongolia/X63/09 was derived from Doroo Tsagaan Nuur in July 2009, at the same location and period as many of the outbreak samples tested in this study were collected. However, despite this anomaly the HI patterns of both these positive control sera were determined to have an HPAIV-bias, and so the impact in the interpretation of field serum samples may have been limited.

This study represents the first serological evidence that relatively large numbers of wild birds are likely surviving exposure to HPAIV H5N1. The implication of this for the spread of HPAIV H5N1 is unclear. Viral challenge that is sufficient to elicit an immune response need not equate to viral replication and shedding. Experimental challenge studies have demonstrated high rates of mortality in some species (such as whooper swans and bar-headed geese [9]), with subclinical infection and virus shedding in others (such as ducks [8]). However, these experimental studies must be interpreted cautiously with respect to the wild situation where exposure to lower doses of virus may enable birds to mount an immune response without incurring infection or subsequent viral release. Further experimental challenge studies have also demonstrated cross-protective immunity to HPAIV H5N1 in birds exposed to other subtypes [37]–[39], which may further enhance the capacity for wild birds to survive HPAIV infection. The protective capacity of antibodies to H5 and heterosubtypic viruses, and the potential impact this could have on herd immunity also warrants closer study. Despite the emergence of two H5N1 clades among wild birds in central Asia, the virus does not appear to persist in the population indefinitely [11], an eventuality that would be hastened by an increase in herd immunity. Alternatively non-protective immunity could actually facilitate the spread of virus, with pre-existing antibody titres enabling survival and viral shedding in species such as swans that are otherwise highly susceptible to infection [40].

Although our findings have important implications for bird populations, the high proportion of sera for which exposure history was deemed ambiguous illustrates the difficulty in using this technique at the individual bird level. Such fine scale interpretation may be advanced through the development of more sophisticated approaches to analyzing data arising from HI analyses. An analytical technique that might be adapted for use in this area is antigenic cartography, which creates a visual representation of antigenic relationships between antigens and antibodies [28]. While this approach is more commonly used to examine antigenic distinctiveness of viruses (e.g. in vaccine selection), it also has potential in profiling the humoral immune response. Successful utilization would require the careful selection of a panel of antigens, with sufficiently distinct antigenic profiles to enable the placement of test sera in a manner that reflected exposure history. Importantly, it would be necessary to verify that the humoral response of different bird species was comparable to prevent anomalies from arising through taxonomic differences.

In conclusion this study has demonstrated the potential of using HI assays to identify populations of wild birds that have been exposed to HPAIV H5N1 strains. This is complicated on an individual bird level by waning titres and repeated infections. However, the technique may have utility in prioritizing populations for HPAIV surveillance and development of risk management measures to prevent exposure of the domestic poultry sector. Further experimental work to determine how the avian immune system responds to repeated exposures of low and highly pathogenic viruses would aid in interpreting future studies of this type, and in understanding the impact of circulating antibodies on population-level immunity and the epidemiology of HPAIV in wild birds.

## Supporting Information

S1 Figure Distribution of domestic and wild bird cases of highly pathogenic avian influenza H5N1 viruses of the Goose/Guangdong/96 H5 lineage reported between January 2004 and February 2012.
(PDF)

S2 Figure Wintering distribution of waterfowl captured in Mongolia.
(PDF)

S3 Figure Distribution of waterfowl sampled in Europe.
(PDF)

S1 Table The results of hemagglutinin inhibition assays to detect antibody titres in serum samples collected from Mongolian wild birds against a panel of six standard influenza A virus H5 antigens.
(DOCX)

S2 Table The results of hemagglutinin inhibition assays to detect antibody titres in serum samples collected from European wild birds against a panel of six standard influenza A virus H5 antigens.
(DOCX)

S3 Table The results of hemagglutinin inhibition to detect antibody titres in serum samples from wild birds used as positive controls against a panel of six standard influenza A virus H5 antigens.
(DOCX)

S4 Table The results of hemagglutinin inhibition to detect antibody titres in ferret antisera raised through exposure to four Classical H5 lineage avian influenza viruses, against twelve Classical H5 lineage avian influenza viruses, and five highly pathogenic avian influenza H5N1 viruses of the Goose/Guangdong/96 H5 lineage.
(DOCX)
