# Supplementary material for: Serological Evidence for Non-Lethal Exposures of Mongolian Wild Birds to Highly Pathogenic Avian Influenza H5N1 Virus
Source: PLoS One. 2014 Dec 15;9(12):e113569. doi: 10.1371/journal.pone.0113569 (PMC4266605; doi:10.1371/journal.pone.0113569)
Supplement: S1 Figure — Distribution of domestic and wild bird cases of highly pathogenic avian influenza H5N1 viruses of the Goose/Guangdong/96 H5 lineage reported between January 2004 and February 2012. (PDF) [file pone.0113569.s001.pdf]

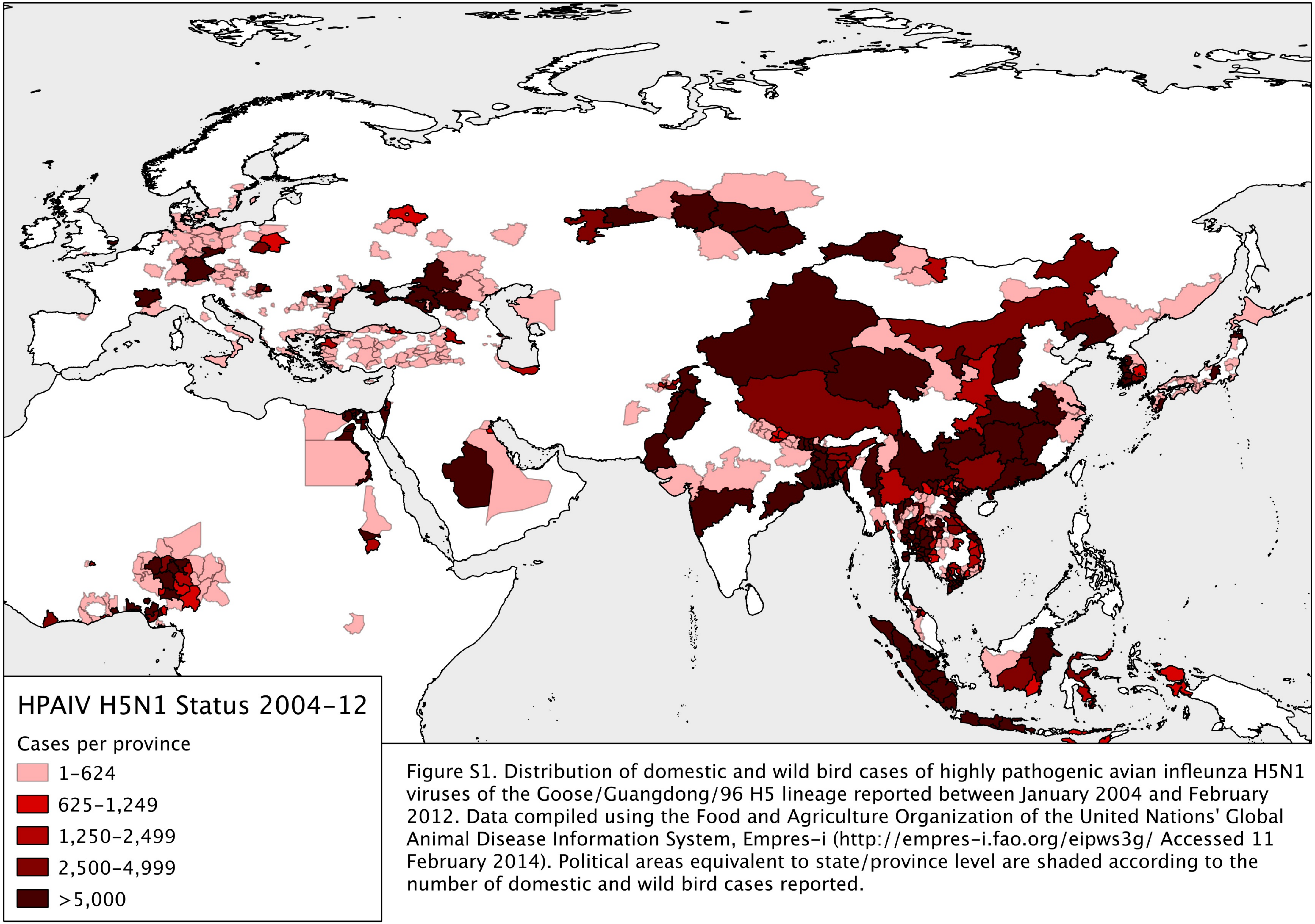

Figure S1. Distribution of domestic and wild bird cases of highly pathogenic avian infleunza H5N1 viruses of the Goose/Guangdong/96 H5 lineage reported between January 2004 and February 2012. Data compiled using the Food and Agriculture Organization of the United Nations' Global Animal Disease Information System, Empres-i (http://empres-i.fao.org/eipws3g/ Accessed 11 February 2014). Political areas equivalent to state/province level are shaded according to the number of domestic and wild bird cases reported.
