# Supplementary material for: Serological Evidence for Non-Lethal Exposures of Mongolian Wild Birds to Highly Pathogenic Avian Influenza H5N1 Virus
Source: PLoS One. 2014 Dec 15;9(12):e113569. doi: 10.1371/journal.pone.0113569 (PMC4266605; doi:10.1371/journal.pone.0113569)
Supplement: S2 Figure — Wintering distribution of waterfowl captured in Mongolia. (PDF) [file pone.0113569.s002.pdf]

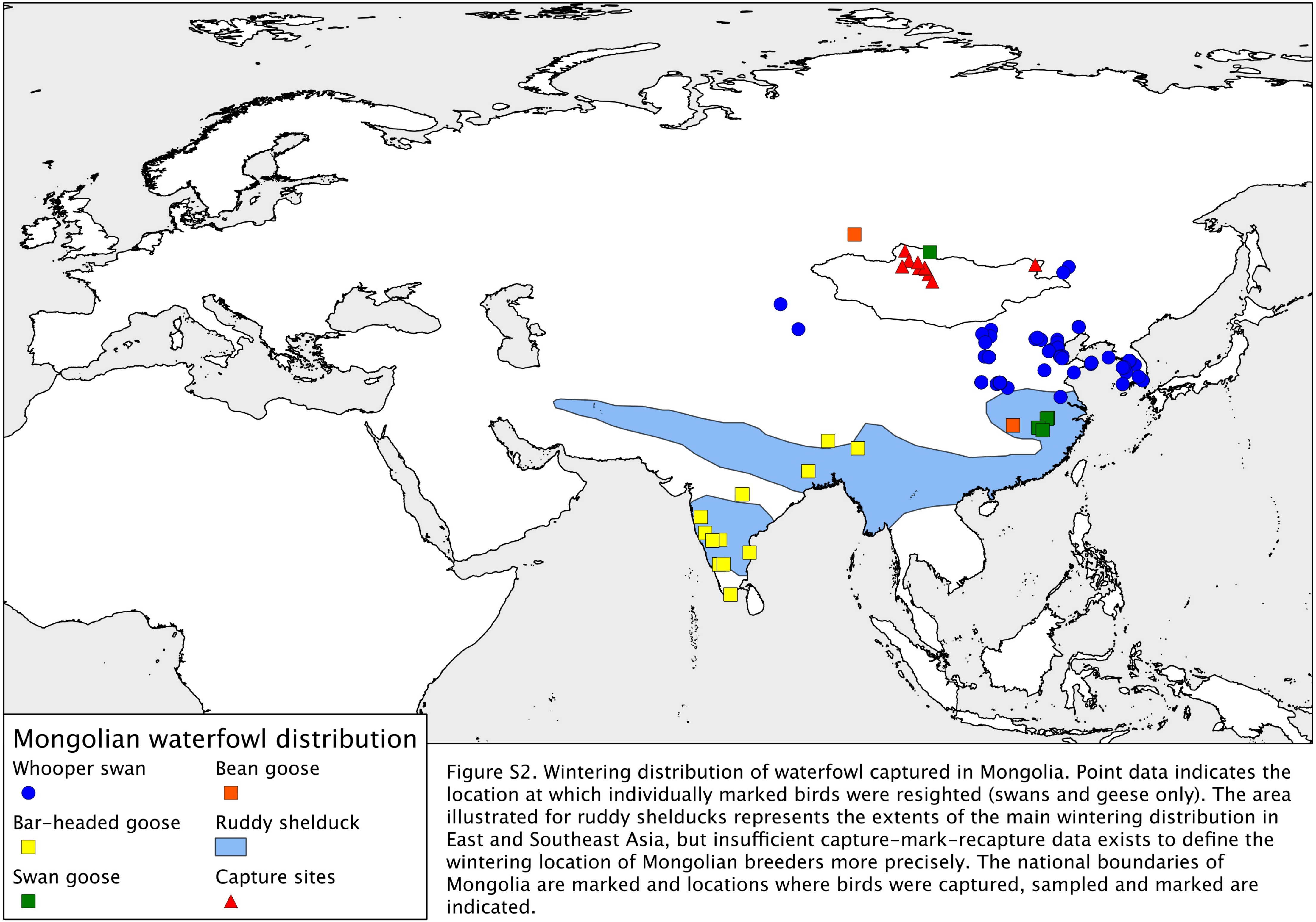

Figure S2. Wintering distribution of waterfowl captured in Mongolia. Point data indicates the location at which individually marked birds were resighted (swans and geese only). The area illustrated for ruddy shelducks represents the extents of the main wintering distribution in East and Southeast Asia, but insufficient capture–mark–recapture data exists to define the wintering location of Mongolian breeders more precisely. The national boundaries of Mongolia are marked and locations where birds were captured, sampled and marked are indicated.
