# Supplementary material for: Serological Evidence for Non-Lethal Exposures of Mongolian Wild Birds to Highly Pathogenic Avian Influenza H5N1 Virus
Source: PLoS One. 2014 Dec 15;9(12):e113569. doi: 10.1371/journal.pone.0113569 (PMC4266605; doi:10.1371/journal.pone.0113569)
Supplement: S3 Figure — Distribution of waterfowl sampled in Europe. (PDF) [file pone.0113569.s003.pdf]

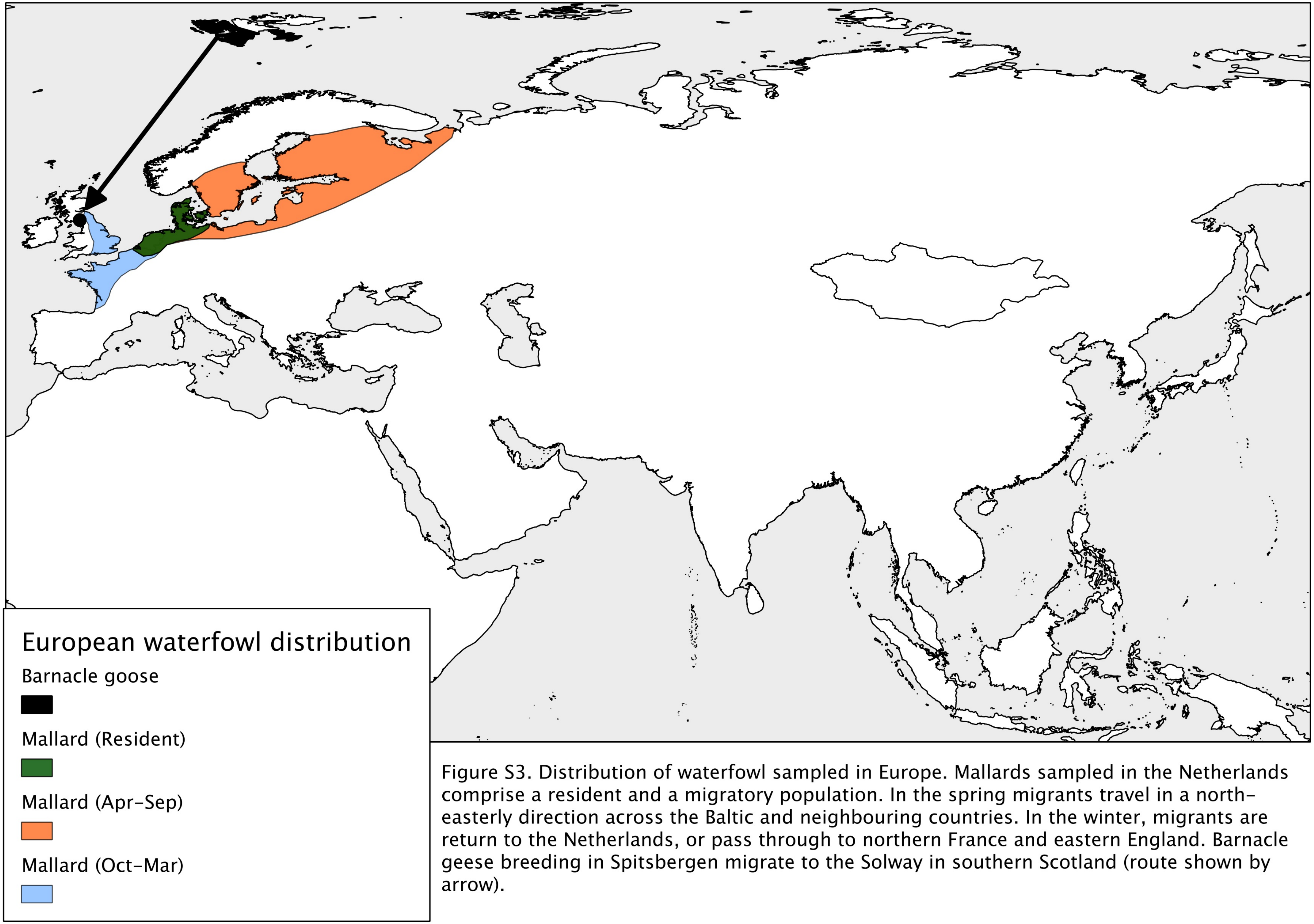

Figure S3. Distribution of waterfowl sampled in Europe. Mallards sampled in the Netherlands comprise a resident and a migratory population. In the spring migrants travel in a north-easterly direction across the Baltic and neighbouring countries. In the winter, migrants are return to the Netherlands, or pass through to northern France and eastern England. Barnacle geese breeding in Spitsbergen migrate to the Solway in southern Scotland (route shown by arrow).
