# Supplementary material for: Serological Evidence for Non-Lethal Exposures of Mongolian Wild Birds to Highly Pathogenic Avian Influenza H5N1 Virus
Source: PLoS One. 2014 Dec 15;9(12):e113569. doi: 10.1371/journal.pone.0113569 (PMC4266605; doi:10.1371/journal.pone.0113569)
Supplement: S1 Table — The results of hemagglutinin inhibition assays to detect antibody titres in serum samples collected from Mongolian wild birds against a panel of six standard influenza A virus H5 antigens. (DOCX) [file pone.0113569.s004.docx]

Table S1. The results of hemagglutinin inhibition assays to detect antibody titres in serum samples collected from Mongolian wild birds against a panel of six standard influenza A virus H5 antigens, including A/mallard/Netherlands/3/99(H5N2) (NL99), A/HongKong/156/97(H5N1) (HK97), A/Viet Nam/1194/2004(H5N1) VN04, A/Indonesia/5/2005(H5N1) (ID05), A/turkey/Turkey/1/2005(H5N1) (TU05), and A/Anhui/1/2005(H5N1) (AN05). Date of sample collection is provided in DD/MM/YY format, and place in WGS84 (latitude, longitude). The health status of each bird is noted as either healthy (H), or sick (S).

|  |  |  |  |  |  | **H5 virus name (clade)** | | | | | |
| --- | --- | --- | --- | --- | --- | --- | --- | --- | --- | --- | --- |
| **Sample code** | **Species name** | **Date** | **Place**  **(N, E)** | **Health status** | **Outbreak** | **NL99 (ClassicClade)** | **HK97 (Clade 0)** | **VN04**  **(Clade 1)** | **ID05**  **(Clade 2.1)** | **TU05**  **(Clade 2.2)** | **AN05**  **(Clade 2.3)** |
| MN08 A-0001 | Tufted duck | 14/05/08 | 49.97, 99.91 | H | No | 20 | 60 | <10 | 160 | 120 | 80 |
| MN08 A-0003 | Northern shoveler | 14/05/08 | 49.26, 99.07 | H | No | <10 | 30 | <10 | <10 | <10 | <10 |
| MN08 A-0010 | Bean goose | 01/07/08 | 49.26, 99.07 | H | No | <10 | 10 | <10 | <10 | <10 | <10 |
| MN08 A-0011 | Bean goose | 01/07/08 | 49.26, 99.07 | H | No | <10 | 30 | <10 | <10 | <10 | <10 |
| MN08 A-0014 | Common merganser | 01/07/08 | 49.26, 99.07 | H | No | 10 | 40 | <10 | <10 | 20 | <10 |
| MN08 A-0016 | Bean goose | 02/07/08 | 49.26, 99.07 | H | No | <10 | 20 | <10 | <10 | <10 | <10 |
| MN08 A-0019 | Bean goose | 02/07/08 | 49.26, 99.07 | H | No | 10 | 40 | <10 | <10 | <10 | <10 |
| MN08 A-0034 | Bean goose | 03/07/08 | 49.26, 99.07 | H | No | <10 | 20 | <10 | <10 | <10 | <10 |
| MN08 A-0036 | Bean goose | 03/07/08 | 49.26, 99.07 | H | No | <10 | 30 | <10 | <10 | <10 | <10 |
| MN08 A-0038 | Bean goose | 03/07/08 | 49.26, 99.07 | H | No | <10 | 10 | <10 | <10 | 20 | <10 |
| MN08 A-0043 | Bean goose | 03/07/08 | 49.26, 99.07 | H | No | 10 | 80 | <10 | <10 | <10 | <10 |
| MN08 A-0060 | Bean goose | 04/07/08 | 49.26, 99.07 | H | No | 10 | 20 | <10 | <10 | <10 | <10 |
| MN08 A-0062 | Bean goose | 04/07/08 | 49.26, 99.07 | H | No | 10 | 30 | <10 | <10 | <10 | <10 |
| MN08 A-0065 | Bean goose | 04/07/08 | 48.94, 101.97 | H | No | 240 | 320 | <10 | <10 | <10 | <10 |
| MN08 A-0079 | Whooper swan | 09/07/08 | 48.94, 101.97 | H | No | 20 | 30 | 10 | <10 | 60 | <10 |
| MN08 A-0083 | Whooper swan | 09/07/08 | 48.94, 101.97 | H | No | <10 | 40 | <10 | <10 | 10 | <10 |
| MN08 A-0089 | Whooper swan | 10/07/08 | 48.94, 101.97 | H | No | 30 | 30 | <10 | <10 | <10 | <10 |
| MN08 A-0111 | Whooper swan | 11/07/08 | 48.94, 101.97 | H | No | <10 | 40 | <10 | <10 | 10 | <10 |
| MN08 A-0112 | Whooper swan | 11/07/08 | 48.94, 101.97 | H | No | <10 | 20 | <10 | <10 | <10 | 10 |
| MN08 A-0114 | Whooper swan | 11/07/08 | 48.94, 101.97 | H | No | 20 | 40 | <10 | <10 | <10 | <10 |
| MN08 A-0119 | Bar-headed goose | 12/07/08 | 48.94, 101.97 | H | No | 160 | 160 | 20 | 30 | 320 | 20 |
| MN08 A-0136 | Bar-headed goose | 12/07/08 | 48.94, 101.97 | H | No | 80 | 160 | 10 | <10 | 160 | 20 |
| MN08 A-0149 | Bar-headed goose | 13/07/08 | 48.94, 101.97 | H | No | <10 | 20 | <10 | <10 | <10 | <10 |
| MN08 A-0159 | Bar-headed goose | 13/07/08 | 48.94, 101.97 | H | No | <10 | 10 | <10 | <10 | 20 | <10 |
| MN08 A-0161 | Bar-headed goose | 13/07/08 | 48.94, 101.97 | H | No | 20 | 60 | <10 | <10 | 40 | <10 |
| MN08 A-0178 | Bar-headed goose | 13/07/08 | 51.2, 99.41 | H | No | <10 | 20 | <10 | <10 | 30 | 20 |
| MN08 A-0227 | Bar-headed goose | 17/07/08 | 51.2, 99.41 | H | No | <10 | 30 | <10 | <10 | 20 | <10 |
| MN08 A-0258 | Bar-headed goose | 17/07/08 | 51.2, 99.41 | H | No | 40 | 40 | <10 | <10 | 40 | <10 |
| MN08 A-0262 | Bar-headed goose | 17/07/08 | 51.2, 99.41 | H | No | <10 | <10 | <10 | <10 | 20 | <10 |
| MN08 A-0298 | Bar-headed goose | 19/07/08 | 49.97, 99.91 | H | No | 60 | 80 | <10 | <10 | 60 | 40 |
| MN08 A-0312 | Whooper swan | 21/07/08 | 49.97, 99.91 | H | No | 20 | 40 | <10 | <10 | 20 | <10 |
| MN08 A-0324 | Whooper swan | 21/07/08 | 49.97, 99.91 | H | No | 10 | 20 | <10 | <10 | 40 | <10 |
| MN08 A-0328 | Whooper swan | 21/07/08 | 49.97, 99.91 | H | No | 160 | 320 | 40 | 20 | 30 | 30 |
| MN08 A-0337 | Whooper swan | 21/07/08 | 49.97, 99.91 | H | No | <10 | 20 | <10 | <10 | <10 | 20 |
| MN08 A-0340 | Whooper swan | 21/07/08 | 49.97, 99.91 | H | No | <10 | 40 | <10 | <10 | <10 | <10 |
| MN08 A-0346 | Whooper swan | 21/07/08 | 49.97, 99.91 | H | No | 20 | 30 | 20 | <10 | 40 | 20 |
| MN08 A-0348 | Whooper swan | 21/07/08 | 49.97, 99.91 | H | No | 20 | 60 | <10 | <10 | 20 | 10 |
| MN08 A-0350 | Bar-headed goose | 22/07/08 | 49.97, 99.91 | H | No | 30 | 160 | 20 | <10 | 40 | <10 |
| MN08 A-0354 | Bar-headed goose | 22/07/08 | 49.97, 99.91 | H | No | 60 | 30 | <10 | <10 | <10 | <10 |
| MN08 A-0359 | Bar-headed goose | 22/07/08 | 49.97, 99.91 | H | No | <10 | 20 | <10 | <10 | <10 | <10 |
| MN08 A-0378 | Bar-headed goose | 22/07/08 | 49.97, 99.91 | H | No | <10 | 20 | <10 | <10 | <10 | <10 |
| MN08 A-0383 | Bar-headed goose | 22/07/08 | 49.97, 99.91 | H | No | <10 | 30 | <10 | <10 | 30 | <10 |
| MN08 A-0396 | Bar-headed goose | 22/07/08 | 49.97, 99.91 | H | No | 30 | 160 | <10 | 20 | 160 | 40 |
| MN08 A-0411 | Bar-headed goose | 22/07/08 | 49.97, 99.91 | H | No | <10 | 80 | <10 | <10 | <10 | <10 |
| MN08 A-0414 | Bar-headed goose | 22/07/08 | 49.97, 99.91 | H | No | <10 | 10 | <10 | <10 | <10 | <10 |
| MN08 A-0418 | Bar-headed goose | 22/07/08 | 49.97, 99.91 | H | No | <10 | 40 | <10 | <10 | 30 | <10 |
| MN08 A-0420 | Bar-headed goose | 22/07/08 | 49.97, 99.91 | H | No | 10 | 120 | <10 | <10 | 80 | 20 |
| MN08 A-0423 | Bar-headed goose | 22/07/08 | 49.26, 99.07 | H | No | <10 | <10 | <10 | <10 | 40 | <10 |
| MN08 A-0467 | Bean goose | 24/07/08 | 49.26, 99.07 | H | No | 60 | 320 | 10 | <10 | 30 | 40 |
| MN08 A-0469 | Bean goose | 24/07/08 | 48.94, 101.97 | H | No | <10 | 30 | <10 | <10 | <10 | <10 |
| MN08 A-0571 | Swan goose | 29/07/08 | 49.77, 101.01 | H | No | <10 | 20 | <10 | <10 | <10 | <10 |
| MN08 A-0672 | Ruddy shelduck | 04/08/08 | 49.77, 101.01 | H | No | <10 | 10 | <10 | <10 | <10 | <10 |
| MN08 A-0674 | Ruddy shelduck | 04/08/08 | 49.77, 101.01 | H | No | <10 | 60 | <10 | <10 | <10 | <10 |
| MN08 A-0679 | Ruddy shelduck | 04/08/08 | 49.77, 101.01 | H | No | 80 | 160 | 20 | 60 | 80 | 60 |
| MN08 A-0683 | Ruddy shelduck | 04/08/08 | 49.77, 101.01 | H | No | <10 | 30 | <10 | <10 | <10 | <10 |
| MN08 A-0685 | Ruddy shelduck | 04/08/08 | 49.77, 101.01 | H | No | <10 | <10 | <10 | <10 | <10 | 20 |
| MN08 A-0687 | Ruddy shelduck | 04/08/08 | 49.77, 101.01 | H | No | 30 | 160 | 20 | 30 | 40 | 30 |
| MN08 A-0689 | Ruddy shelduck | 04/08/08 | 49.77, 101.01 | H | No | 20 | 40 | 10 | <10 | 30 | <10 |
| MN08 A-0698 | Ruddy shelduck | 04/08/08 | 49.77, 101.01 | H | No | <10 | 40 | <10 | <10 | <10 | <10 |
| MN08 A-0699 | Ruddy shelduck | 04/08/08 | 49.77, 101.01 | H | No | <10 | 10 | <10 | <10 | <10 | <10 |
| MN08 A-0705 | Ruddy shelduck | 04/08/08 | 49.77, 101.01 | H | No | <10 | 40 | <10 | <10 | <10 | <10 |
| MN08 A-0711 | Ruddy shelduck | 05/08/08 | 49.77, 101.01 | H | No | <10 | <10 | 10 | 30 | 10 | 30 |
| MN08 A-0717 | Ruddy shelduck | 05/08/08 | 49.77, 101.01 | H | No | 40 | 80 | 20 | 40 | 30 | 40 |
| MN08 A-0718 | Ruddy shelduck | 05/08/08 | 49.77, 101.01 | H | No | 160 | 320 | 40 | 160 | 160 | 80 |
| MN08 A-0720 | Ruddy shelduck | 05/08/08 | 49.77, 101.01 | H | No | <10 | 80 | <10 | <10 | <10 | 40 |
| MN08 A-0725 | Ruddy shelduck | 05/08/08 | 49.77, 101.01 | H | No | <10 | 20 | <10 | <10 | <10 | <10 |
| MN08 A-0728 | Ruddy shelduck | 05/08/08 | 49.77, 101.01 | H | No | <10 | 20 | <10 | <10 | <10 | <10 |
| MN08 A-0736 | Ruddy shelduck | 05/08/08 | 49.77, 101.01 | H | No | 30 | 120 | <10 | 30 | 40 | 40 |
| MN08 A-0746 | Ruddy shelduck | 05/08/08 | 49.77, 101.01 | H | No | <10 | 40 | <10 | <10 | <10 | <10 |
| MN08 A-0748 | Ruddy shelduck | 05/08/08 | 49.77, 101.01 | H | No | 80 | 160 | 40 | 40 | 80 | 80 |
| MN08 A-0754 | Ruddy shelduck | 05/08/08 | 49.77, 101.01 | H | No | 240 | 320 | 20 | 60 | 60 | 40 |
| MN08 A-0755 | Ruddy shelduck | 05/08/08 | 49.77, 101.01 | H | No | <10 | 10 | <10 | <10 | <10 | <10 |
| MN08 A-0757 | Ruddy shelduck | 05/08/08 | 49.77, 101.01 | H | No | <10 | 20 | <10 | <10 | <10 | <10 |
| MN08 A-0767 | Ruddy shelduck | 06/08/08 | 49.77, 101.01 | H | No | 160 | 160 | 80 | 60 | 80 | 320 |
| MN08 A-0769 | Ruddy shelduck | 06/08/08 | 49.77, 101.01 | H | No | 60 | 40 | 20 | 30 | 40 | 20 |
| MN08 A-0770 | Ruddy shelduck | 06/08/08 | 49.77, 101.01 | H | No | 80 | 160 | 60 | 60 | 120 | 120 |
| MN08 A-0773 | Ruddy shelduck | 06/08/08 | 49.77, 101.01 | H | No | 80 | 160 | 30 | 20 | 60 | 20 |
| MN08 A-0775 | Ruddy shelduck | 06/08/08 | 49.77, 101.01 | H | No | 80 | 160 | 30 | 30 | 40 | 80 |
| MN08 A-0776 | Ruddy shelduck | 06/08/08 | 49.77, 101.01 | H | No | 60 | 80 | 20 | 40 | 40 | 20 |
| MN08 A-0781 | Ruddy shelduck | 06/08/08 | 49.77, 101.01 | H | No | 30 | 30 | <10 | <10 | 10 | <10 |
| MN08 A-0789 | Ruddy shelduck | 06/08/08 | 49.77, 101.01 | H | No | <10 | 30 | <10 | <10 | <10 | <10 |
| MN08 A-0796 | Ruddy shelduck | 06/08/08 | 49.77, 101.01 | H | No | <10 | 30 | <10 | <10 | <10 | <10 |
| MN08 A-0797 | Ruddy shelduck | 06/08/08 | 49.77, 101.01 | H | No | <10 | 30 | <10 | <10 | <10 | <10 |
| MN08 A-0798 | Ruddy shelduck | 06/08/08 | 49.77, 101.01 | H | No | <10 | 10 | <10 | <10 | <10 | <10 |
| MN08 A-0799 | Ruddy shelduck | 06/08/08 | 49.77, 101.01 | H | No | 240 | 320 | 80 | 240 | 160 | 80 |
| MN08 A-0800 | Ruddy shelduck | 06/08/08 | 49.77, 101.01 | H | No | <10 | 20 | <10 | <10 | <10 | <10 |
| MN08 A-0801 | Ruddy shelduck | 06/08/08 | 49.77, 101.01 | H | No | 240 | 320 | 80 | 320 | 160 | 160 |
| MN08 A-0802 | Ruddy shelduck | 06/08/08 | 49.77, 101.01 | H | No | 20 | 40 | <10 | <10 | <10 | <10 |
| MN08 A-0806 | Ruddy shelduck | 06/08/08 | 49.77, 101.01 | H | No | 60 | 40 | <10 | 20 | 40 | <10 |
| MN08 A-0807 | Ruddy shelduck | 06/08/08 | 49.77, 101.01 | H | No | <10 | <10 | <10 | 80 | <10 | 30 |
| MN08 A-0808 | Ruddy shelduck | 06/08/08 | 49.77, 101.01 | H | No | 240 | 240 | 40 | 120 | 160 | 40 |
| MN08 A-0810 | Ruddy shelduck | 06/08/08 | 49.77, 101.01 | H | No | 20 | 20 | <10 | 20 | <10 | <10 |
| MN08 A-0811 | Ruddy shelduck | 06/08/08 | 49.77, 101.01 | H | No | 60 | 120 | <60 | <60 | <60 | <60 |
| MN08 A-0814 | Ruddy shelduck | 06/08/08 | 49.77, 101.01 | H | No | 10 | 40 | <10 | <10 | <10 | <10 |
| MN08 A-0828 | Ruddy shelduck | 06/08/08 | 49.77, 101.01 | H | No | <10 | 40 | <10 | <10 | <10 | <10 |
| MN08 A-0834 | Ruddy shelduck | 06/08/08 | 50.52, 100.39 | H | No | 320 | 480 | 80 | 320 | 320 | 160 |
| MN08 A-0998 | Mongolian gull | 12/08/08 | 49.77, 101.01 | H | No | 20 | 30 | <10 | <10 | <10 | <10 |
| MN09 A-0009 | Whooper swan | 04/07/09 | 49.77, 101.01 | H | No | <10 | 30 | <10 | <10 | <10 | <10 |
| MN09 A-0010 | Whooper swan | 04/07/09 | 51.2, 99.41 | H | No | 480 | 960 | 30 | 240 | 480 | 640 |
| MN09 A-0034 | Bar-headed goose | 08/07/09 | 51.2, 99.41 | H | No | 30 | 60 | 20 | <10 | 80 | <10 |
| MN09 A-0060 | Bar-headed goose | 10/07/09 | 51.2, 99.41 | H | No | <10 | 10 | <10 | <10 | 20 | <10 |
| MN09 A-0100 | Bar-headed goose | 10/07/09 | 49.97, 99.91 | H | No | 20 | 60 | <10 | 20 | 40 | 30 |
| MN09 A-0103 | Tundra swan | 12/07/09 | 48.94, 101.97 | S | No | 480 | 1280 | 160 | 120 | 320 | 480 |
| MN09 A-0145 | Bar-headed goose | 14/07/09 | 48.94, 101.97 | H | No | 20 | <10 | <10 | <10 | <10 | <10 |
| MN09 A-0157 | Bar-headed goose | 14/07/09 | 48.94, 101.97 | H | No | <10 | 40 | <10 | <10 | <10 | <10 |
| MN09 A-0161 | Bar-headed goose | 14/07/09 | 48.94, 101.97 | H | No | 20 | 40 | <10 | <10 | 30 | <10 |
| MN09 A-0166 | Bar-headed goose | 14/07/09 | 48.94, 101.97 | H | No | <10 | <10 | <10 | <10 | 20 | <10 |
| MN09 A-0167 | Bar-headed goose | 14/07/09 | 48.94, 101.97 | H | No | <10 | 20 | <10 | <10 | <10 | <10 |
| MN09 A-0177 | Bar-headed goose | 14/07/09 | 48.94, 101.97 | H | No | <10 | 20 | <10 | <10 | 30 | <10 |
| MN09 A-0181 | Bar-headed goose | 14/07/09 | 48.94, 101.97 | H | No | <10 | 30 | <10 | <10 | <10 | <10 |
| MN09 A-0238 | Whooper swan | 15/07/09 | 48.94, 101.97 | H | No | 30 | 80 | <10 | 20 | 30 | 30 |
| MN09 A-0240 | Whooper swan | 15/07/09 | 48.94, 101.97 | H | No | <10 | 20 | <10 | <10 | <10 | <10 |
| MN09 A-0242 | Bar-headed goose | 15/07/09 | 48.94, 101.97 | H | No | <10 | 10 | <10 | <10 | 40 | <10 |
| MN09 A-0243 | Swan goose | 15/07/09 | 48.94, 101.97 | H | No | <10 | 40 | <10 | <10 | 40 | <10 |
| MN09 A-0251 | Bar-headed goose | 16/07/09 | 48.28, 102.34 | H | No | 80 | 320 | 20 | 40 | 60 | 60 |
| MN09 A-0302 | Whooper swan | 19/07/09 | 48.28, 102.34 | H | No | <10 | 30 | <10 | <10 | <10 | <10 |
| MN09 A-0303 | Whooper swan | 19/07/09 | 47.36, 102.81 | H | No | 30 | 120 | <10 | <10 | 20 | <10 |
| MN09 A-0399 | Swan goose | 23/07/09 | 48.28, 102.34 | H | No | <10 | 30 | <10 | <10 | 20 | <10 |
| MN09 A-0411 | Bar-headed goose | 24/07/09 | 48.28, 102.34 | H | No | 10 | 20 | <10 | <10 | 10 | <10 |
| MN09 A-0450 | Bar-headed goose | 24/07/09 | 48.94, 101.97 | H | No | <10 | 10 | <10 | <10 | <10 | <10 |
| MN09 A-0544 | Whooper swan | 26/07/09 | 49.97, 99.91 | H | No | <10 | 20 | <10 | <10 | <10 | <10 |
| MN09 A-0551 | Whooper swan | 28/07/09 | 48.94, 101.97 | H | Yes | 10 | <10 | <10 | 60 | 160 | 30 |
| MN09 A-0552 | Whooper swan | 26/07/09 | 49.97, 99.91 | H | No | <10 | 10 | <10 | <10 | <10 | <10 |
| MN09 A-0556 | Ruddy shelduck | 28/07/09 | 49.97, 99.91 | S | Yes | <10 | 80 | <10 | 20 | <10 | <10 |
| MN09 A-0576 | Ruddy shelduck | 29/07/09 | 49.97, 99.91 | H | Yes | 20 | 120 | <10 | 40 | 40 | <10 |
| MN09 A-0580 | Ruddy shelduck | 29/07/09 | 49.97, 99.91 | H | Yes | <10 | 160 | <10 | <10 | 10 | <10 |
| MN09 A-0582 | Ruddy shelduck | 29/07/09 | 49.97, 99.91 | H | Yes | 160 | 320 | 20 | 120 | 80 | 40 |
| MN09 A-0586 | Bean goose | 29/07/09 | 49.97, 99.91 | H | Yes | <10 | 30 | <10 | <10 | <10 | 80 |
| MN09 A-0592 | Ruddy shelduck | 29/07/09 | 49.97, 99.91 | H | Yes | <10 | 10 | <10 | <10 | <10 | <10 |
| MN09 A-0593 | Ruddy shelduck | 29/07/09 | 49.97, 99.91 | H | Yes | 10 | 40 | <10 | 10 | 10 | <10 |
| MN09 A-0594 | Ruddy shelduck | 29/07/09 | 49.97, 99.91 | H | Yes | 80 | 120 | 40 | 80 | 80 | 20 |
| MN09 A-0609 | Ruddy shelduck | 29/07/09 | 49.97, 99.91 | H | Yes | 160 | 160 | 40 | 80 | 80 | 40 |
| MN09 A-0615 | Ruddy shelduck | 29/07/09 | 49.97, 99.91 | H | Yes | 30 | 80 | <10 | 30 | 40 | 40 |
| MN09 A-0617 | Ruddy shelduck | 30/07/09 | 49.97, 99.91 | H | Yes | <10 | 20 | <10 | <10 | <10 | <10 |
| MN09 A-0627 | Ruddy shelduck | 30/07/09 | 49.97, 99.91 | H | Yes | <10 | 10 | <10 | <10 | <10 | <10 |
| MN09 A-0629 | Ruddy shelduck | 30/07/09 | 49.97, 99.91 | H | Yes | 120 | 120 | 20 | 80 | 80 | 40 |
| MN09 A-0636 | Ruddy shelduck | 30/07/09 | 49.97, 99.91 | H | Yes | <10 | 20 | <10 | <10 | <10 | <10 |
| MN09 A-0648 | Ruddy shelduck | 30/07/09 | 49.97, 99.91 | H | Yes | <10 | 20 | <10 | <10 | <10 | <10 |
| MN09 A-0651 | Ruddy shelduck | 30/07/09 | 49.97, 99.91 | H | Yes | <10 | 30 | <10 | <10 | <10 | <10 |
| MN09 A-0658 | Ruddy shelduck | 30/07/09 | 49.97, 99.91 | H | Yes | <10 | 40 | <10 | <10 | <10 | <10 |
| MN09 A-0659 | Ruddy shelduck | 30/07/09 | 49.97, 99.91 | H | Yes | <10 | 40 | <10 | <10 | <10 | <10 |
| MN09 A-0667 | Ruddy shelduck | 30/07/09 | 49.97, 99.91 | H | Yes | 40 | 120 | 20 | 20 | 40 | 20 |
| MN09 A-0668 | Ruddy shelduck | 30/07/09 | 49.97, 99.91 | H | Yes | 160 | 240 | 80 | 640 | 160 | 160 |
| MN09 A-0673 | Ruddy shelduck | 30/07/09 | 49.97, 99.91 | H | Yes | <10 | <10 | <10 | 10 | <10 | <10 |
| MN09 A-0674 | Ruddy shelduck | 30/07/09 | 49.97, 99.91 | H | Yes | <10 | 20 | <10 | 10 | <10 | <10 |
| MN09 A-0676 | Ruddy shelduck | 30/07/09 | 49.97, 99.91 | H | Yes | 20 | 60 | <10 | <10 | 20 | <10 |
| MN09 A-0689 | Ruddy shelduck | 31/07/09 | 49.97, 99.91 | H | Yes | <10 | 20 | <10 | <10 | <10 | <10 |
| MN09 A-0693 | Ruddy shelduck | 31/07/09 | 49.97, 99.91 | H | Yes | 20 | 60 | <10 | <10 | 10 | <10 |
| MN09 A-0699 | Ruddy shelduck | 31/07/09 | 49.97, 99.91 | H | Yes | <10 | 20 | <10 | <10 | <10 | <10 |
| MN09 A-0716 | Ruddy shelduck | 31/07/09 | 49.97, 99.91 | H | Yes | 20 | 40 | <10 | 10 | <10 | <10 |
| MN09 A-0719 | Bar-headed goose | 31/07/09 | 49.97, 99.91 | H | Yes | <10 | <10 | <10 | <10 | 20 | <10 |
| MN09 A-0720 | Bar-headed goose | 31/07/09 | 49.97, 99.91 | H | Yes | <10 | 60 | <10 | 160 | 80 | 40 |
| MN09 A-0723 | Ruddy shelduck | 31/07/09 | 49.97, 99.91 | H | Yes | 20 | 30 | <10 | <10 | 20 | <10 |
| MN09 A-0726 | Ruddy shelduck | 31/07/09 | 49.97, 99.91 | H | Yes | <10 | 20 | <10 | <10 | <10 | <10 |
| MN09 A-0729 | Ruddy shelduck | 31/07/09 | 49.97, 99.91 | H | Yes | <10 | 20 | <10 | <10 | <10 | <10 |
| MN09 A-0731 | Ruddy shelduck | 31/07/09 | 49.97, 99.91 | H | Yes | <10 | 30 | <10 | <10 | <10 | <10 |
| MN09 A-0734 | Ruddy shelduck | 31/07/09 | 49.97, 99.91 | H | Yes | 40 | 80 | <10 | 10 | 30 | 10 |
| MN09 A-0735 | Ruddy shelduck | 31/07/09 | 49.97, 99.91 | H | Yes | <10 | 20 | <10 | <10 | <10 | <10 |
| MN09 A-0740 | Ruddy shelduck | 31/07/09 | 49.97, 99.91 | H | Yes | 20 | 60 | <10 | 20 | 30 | 20 |
| MN09 A-0749 | Ruddy shelduck | 31/07/09 | 49.97, 99.91 | H | Yes | <10 | 20 | <10 | <10 | <10 | <10 |
| MN09 A-0750 | Ruddy shelduck | 31/07/09 | 49.77, 101.01 | H | Yes | 30 | 60 | 20 | <10 | 40 | <10 |
| MN09 A-0839 | Ruddy shelduck | 02/08/09 | 49.77, 101.01 | H | No | 40 | 20 | <10 | <10 | 10 | <10 |
| MN09 A-0841 | Ruddy shelduck | 02/08/09 | 49.06, 101.16 | H | No | 10 | 40 | <10 | <10 | 20 | <10 |
| MN09 A-0912 | Bar-headed goose | 06/08/09 | 49.06, 101.16 | H | Yes | <10 | 160 | <10 | 240 | <10 | <10 |
| MN09 A-0915 | Ruddy shelduck | 06/08/09 | 49.06, 101.16 | H | Yes | <10 | 10 | <10 | <10 | <10 | <10 |
| MN09 A-0925 | Ruddy shelduck | 06/08/09 | 49.06, 101.16 | H | Yes | 80 | 120 | 10 | 30 | 80 | 60 |
| MN09 A-0929 | Bar-headed goose | 06/08/09 | 49.06, 101.16 | H | Yes | <10 | <10 | <10 | <10 | 80 | <10 |
| MN09 A-0940 | Ruddy shelduck | 06/08/09 | 49.06, 101.16 | H | Yes | <10 | 20 | <10 | <10 | <10 | <10 |
| MN09 A-0943 | Ruddy shelduck | 06/08/09 | 49.06, 101.16 | H | Yes | <10 | 30 | <10 | <10 | <10 | <10 |
| MN09 A-0952 | Ruddy shelduck | 06/08/09 | 49.06, 101.16 | H | Yes | 40 | 80 | 10 | <10 | 20 | 30 |
| MN09 A-0965 | Ruddy shelduck | 06/08/09 | 49.06, 101.16 | H | Yes | 160 | 160 | 40 | 80 | 80 | 80 |
| MN09 A-0968 | Ruddy shelduck | 06/08/09 | 49.06, 101.16 | H | Yes | 480 | 1280 | 160 | 240 | 240 | 160 |
| MN09 A-0969 | Ruddy shelduck | 07/08/09 | 49.06, 101.16 | S | Yes | 960 | 2560 | 640 | 960 | 960 | 960 |
| MN09 A-0970 | Bar-headed goose | 07/08/09 | 49.06, 101.16 | H | Yes | <10 | 10 | <10 | 10 | 20 | <10 |
| MN09 A-0971 | Bar-headed goose | 07/08/09 | 49.06, 101.16 | H | Yes | <10 | 10 | <10 | 10 | 20 | 20 |
| MN09 A-0974 | Ruddy shelduck | 07/08/09 | 49.06, 101.16 | H | Yes | 40 | 120 | 20 | <10 | 60 | <10 |
| MN09 A-0982 | Ruddy shelduck | 07/08/09 | 49.06, 101.16 | H | Yes | 3840 | 10240 | 1280 | 3840 | 3840 | 5120 |
| MN09 A-0987 | Ruddy shelduck | 07/08/09 | 49.06, 101.16 | H | Yes | 10 | 40 | <10 | <10 | <10 | <10 |
| MN09 A-0988 | Ruddy shelduck | 07/08/09 | 49.06, 101.16 | H | Yes | 2560 | 2560 | 40 | 20 | 1280 | 1280 |
| MN09 A-0993 | Ruddy shelduck | 07/08/09 | 49.06, 101.16 | H | Yes | 20 | 60 | <10 | <10 | 10 | <10 |
| MN09 A-0995 | Ruddy shelduck | 07/08/09 | 49.06, 101.16 | H | Yes | 80 | 160 | 30 | 30 | 60 | 60 |
| MN09 A-1002 | Ruddy shelduck | 07/08/09 | 49.97, 99.91 | H | Yes | 20 | 30 | <10 | <10 | <10 | <10 |
