# Supplementary material for: Serological Evidence for Non-Lethal Exposures of Mongolian Wild Birds to Highly Pathogenic Avian Influenza H5N1 Virus
Source: PLoS One. 2014 Dec 15;9(12):e113569. doi: 10.1371/journal.pone.0113569 (PMC4266605; doi:10.1371/journal.pone.0113569)
Supplement: S2 Table — The results of hemagglutinin inhibition assays to detect antibody titres in serum samples collected from European wild birds against a panel of six standard influenza A virus H5 antigens. (DOCX) [file pone.0113569.s005.docx]

Table S2. The results of hemagglutinin inhibition assays to detect antibody titres in serum samples collected from European wild birds against a panel of six standard influenza A virus H5 antigens, including A/mallard/Netherlands/3/99(H5N2) (NL99), A/HongKong/156/97(H5N1) (HK97), A/Viet Nam/1194/2004(H5N1) VN04, A/Indonesia/5/2005(H5N1) (ID05), A/turkey/Turkey/1/2005(H5N1) (TU05), and A/Anhui/1/2005(H5N1) (AN05). Date of sample collection is provided in DD/MM/YY format, and place in WGS84 (latitude, longitude).

|  |  |  |  | **H5 virus name (clade)** | | | | | |
| --- | --- | --- | --- | --- | --- | --- | --- | --- | --- |
| **Sample code** | **Species name** | **Date** | **Place**  **(N, E)** | **NL99 (Classic)** | **HK97 (Clade 0)** | **VN04**  **(Clade 1)** | **ID05**  **(Clade 2.1)** | **TU05**  **(Clade 2.2)** | **AN05**  **(Clade 2.3)** |
| AF-2 | Mallard | 14/06/11 | 51.97, 4.76 | 640 | 240 | <10 | <10 | <10 | 30 |
| AF-10 | Mallard | 14/06/11 | 51.97, 4.76 | 640 | 320 | 80 | <10 | <10 | 240 |
| AF-22 | Mallard | 14/06/11 | 51.97, 4.76 | 640 | 240 | 80 | 30 | 30 | 320 |
| AF-27 | Mallard | 14/06/11 | 51.97, 4.76 | 640 | 320 | <10 | <10 | 40 | 120 |
| AF-36 | Mallard | 14/06/11 | 51.97, 4.76 | 1280 | 640 | 120 | 60 | 30 | 320 |
| BAGO-21 | Barnacle goose | Jul-Aug '05 | 78.93, 11.93 | 80 | 120 | 20 | 10 | 40 | 20 |
| BAGO-54 | Barnacle goose | Jul-Aug '05 | 78.93, 11.93 | 30 | 30 | <10 | <10 | <10 | <10 |
| BAGO-62 | Barnacle goose | Jul-Aug '05 | 78.93, 11.93 | 160 | 120 | <10 | <10 | 40 | <10 |
| BAGO-70 | Barnacle goose | Jul-Aug '05 | 78.93, 11.93 | 80 | 40 | <10 | 10 | <10 | 20 |
| BAGO-73 | Barnacle goose | Jul-Aug '05 | 78.93, 11.93 | 20 | 30 | <10 | <10 | <10 | <10 |
| BAGO-79 | Barnacle goose | Jul-Aug '05 | 78.93, 11.93 | 20 | 10 | <10 | <10 | <10 | <10 |
| BAGO-100 | Barnacle goose | Jul-Aug '05 | 78.93, 11.93 | 120 | 240 | <10 | <10 | <10 | <10 |
| BAGO-102 | Barnacle goose | Jul-Aug '05 | 78.93, 11.93 | 20 | 40 | <10 | <10 | <10 | <10 |
| BAGO-108 | Barnacle goose | Jul-Aug '05 | 78.93, 11.93 | <10 | 30 | <10 | <10 | <10 | <10 |
| 1A8 | Mallard | 22/2/08 | 52.011, 5.96 | 80 | 160 | 60 | 40 | 40 | 40 |
| 1F9 | Mallard | 22/2/08 | 51.99, 5.90 | 20 | 20 | <10 | <10 | <10 | <10 |
| 2F2 | Mallard | 29/2/08 | 52.57, 5.91 | <10 | 10 | <10 | <10 | 10 | <10 |
| 2A8 | Mallard | 10/3/08 | 52.36, 4.87 | 20 | 30 | <10 | <10 | <10 | <10 |
| 2B8 | Mallard | 10/3/08 | 52.36, 4.87 | <10 | 10 | <10 | <10 | <10 | <10 |
| 255-174 | Greater white-fronted goose | 31/12/11 | 51.80, 5.39 | 60 | 40 | <10 | <10 | <10 | 20 |
| 255-313 | Barnacle goose | 11/1/12 | 53.27, 6.14 | 20 | 10 | <10 | <10 | <10 | <10 |
| 255-342 | Greater white-fronted goose | 14/1/12 | 51.80, 5.44 | 40 | 60 | <10 | <10 | <10 | <10 |
| 3B8 | Egyptian goose | 14/3/08 | 52.36, 4.87 | 20 | 20 | <10 | <10 | 10 | <10 |
| 3D7 | Mallard | 14/3/08 | 52.36, 4.87 | <10 | 10 | <10 | <10 | <10 | <10 |
| 3F7 | Mallard | 14/3/08 | 52.36, 4.87 | 20 | 30 | <10 | 10 | 20 | 20 |
