# Supplementary material for: Serological Evidence for Non-Lethal Exposures of Mongolian Wild Birds to Highly Pathogenic Avian Influenza H5N1 Virus
Source: PLoS One. 2014 Dec 15;9(12):e113569. doi: 10.1371/journal.pone.0113569 (PMC4266605; doi:10.1371/journal.pone.0113569)
Supplement: S3 Table — The results of hemagglutinin inhibition to detect antibody titres in serum samples from wild birds used as positive controls against a panel of six standard influenza A virus H5 antigens. (DOCX) [file pone.0113569.s006.docx]

Table S3. The results of hemagglutinin inhibition to detect antibody titres in serum samples from wild birds used as positive controls against a panel of six standard influenza A virus H5 antigens, including A/mallard/Netherlands/3/99(H5N2) (NL99), A/HongKong/156/97(H5N1) (HK97), A/Viet Nam/1194/2004(H5N1) VN04, A/Indonesia/5/2005(H5N1) (ID05), A/turkey/Turkey/1/2005(H5N1) (TU05), and A/Anhui/1/2005(H5N1) (AN05). Wild birds were all experimentally exposed to subtypes of highly pathogenic avian influenza virus H5N1 (listed). Viral strain names include the acronyms: WS = whooper swan, RS= ruddy shelduck, DK = duck, CK = chicken, AY = Anyang, MN = Mongolia.

|  |  |  |  | **H5 virus name (clade)** | | | | | |
| --- | --- | --- | --- | --- | --- | --- | --- | --- | --- |
| **Sample code** | **Species name** | **Exposure strain** | **Exposure clade** | **NL99 (Classic)** | **HK97 (Clade 0)** | **VN04 (Clade 1)** | **ID05 (Clade 2.1)** | **TU05 (Clade 2.2)** | **AN05 (Clade 2.3)** |
| Bird #134 | Herring gull | A/DK/AY/AVL-1/2001(H5N1) | 1 | <10 | 160 | <10 | <10 | <10 | 40 |
| Bird #143 | Herring gull | A/DK/AY/AVL-1/2001(H5N1) | 1 | <10 | 60 | <10 | <10 | 240 | 20 |
| POCH | Common pochard | A/turkey/Turkey/1/2005(H5N1) | 2.2 | 1280 | 5120 | 640 | 640 | 3840 | 2560 |
| TUDU | Tufted duck | A/turkey/Turkey/1/2005(H5N1) | 2.2 | 240 | 120 | 30 | 640 | 640 | 320 |
| TUPI | Tufted duck | A/turkey/Turkey/1/2005(H5N1) | 2.2 | 1280 | 5120 | 960 | 320 | 1280 | 1920 |
| WIGE | Eurasian wigeon | A/turkey/Turkey/1/2005(H5N1) | 2.2 | 3840 | 20480 | 10240 | 17920 | 40960 | 20480 |
| 256 | Dunlin | A/WS/MN/244/2005(H5N1) | 2.2 | <10 | <10 | <10 | <10 | <10 | <10 |
| 258 | Dunlin | A/WS/MN/244/2005(H5N1) | 2.2 | <10 | 10 | <10 | <10 | <10 | <10 |
| 973 | Dunlin | A/WS/MN/244/2005(H5N1) | 2.2 | <10 | 10 | <10 | <10 | <10 | <10 |
| Bird #44 | Greylag goose | A/CK/Korea/IS/2006(H5N1) | 2.2 | <10 | 60 | <10 | <10 | <10 | <10 |
| Bird #43 | Greylag goose | A/CK/Korea/IS/2006(H5N1) | 2.2 | <10 | 640 | 640 | <10 | 960 | 80 |
| Bird #10 | Mandarin duck | A/CK/Korea/IS/2006(H5N1) | 2.2 | <10 | 10 | <10 | <10 | 20 | <10 |
| Bird #25 | Bar-headed goose | A/WS/MN/244/2005(H5N1) | 2.2 | <10 | 160 | <10 | <10 | 60 | <10 |
| Bird #81 | Bar-headed goose | A/RS/MN/X63/2009(H5N1) | 2.3 | <10 | 30 | <10 | <10 | 40 | <10 |
| Bird #94 | Bar-headed goose | A/RS/MN/X63/2009(H5N1) | 2.3 | <10 | 80 | <10 | 60 | 20 | <10 |
