# Supplementary material for: Serological Evidence for Non-Lethal Exposures of Mongolian Wild Birds to Highly Pathogenic Avian Influenza H5N1 Virus
Source: PLoS One. 2014 Dec 15;9(12):e113569. doi: 10.1371/journal.pone.0113569 (PMC4266605; doi:10.1371/journal.pone.0113569)
Supplement: S4 Table — The results of hemagglutinin inhibition to detect antibody titres in ferret antisera raised through exposure to four Classical H5 lineage avian influenza viruses, against twelve Classical H5 lineage avian influenza viruses, and five highly pathogenic avian influenza H5N1 viruses of the Goose/Guangdong/96 H5 lineage. (DOCX) [file pone.0113569.s007.docx]

Table S4. Ferret antisera were raised against four influenza A viruses within the Classical H5 lineage: A/Tern/SouthAfrica/1963 (TE/SA/63), A/Duck/HongKong/205/1977 (DK/HK/77), A/Mallard/Netherlands/3/1999 (MA/NL/99), A/Mallard/Sweden/718/2002 (MA/SE/02). These antisera were then used to measure hemagglutination inhibition (HI) antibody titres against twelve viruses from the Classical H5 lineage, and five viruses within the Asian H5 lineage A/Goose/Guangdong/1/96 (including clades 0, 1, 2.1, 2.2 and 2.3). The HI titers provided represent mean values from 2 to 5 individual HI tables. Titres against homologous viruses are indicated in bold underlined font. This table shows that low pathogenic avian influenza (LPAI) viruses isolated over multiple years (1959 - 2002) and from a range of locations were antigenically similar. This table further shows that ferret antisera raised against LPAI viruses are "LPAI-biased" i.e. have higher titers to LPAI strains than to highly pathogenic avian influenza (HPAI) strains. The table provides further support for excluding A/Hong Kong/156/1997 from the analyses, as this strain yields high HI titers to all sera, regardless whether they were raised against HPAI or LPAI viruses.

| **Virus** | **Virus**  **lineage** | **Ferret antisera** | | | |
| --- | --- | --- | --- | --- | --- |
|  |  | **TE/SA/63** | **DK/HK/77** | **MA/NL/99** | **MA/SE/02** |
|  |  |  |  |  |  |
| A/CHICKEN/SCOTLAND/1959 | Classical | 560 | 2880 | 80 | 320 |
| A/TERN/SOUTHAFRICA/1963 | Classical | **400** | 1280 | 15 | 80 |
| A/TURKEY/WISCONSIN/1968 | Classical | 480 | 800 | 60 | <10 |
| A/DUCK/HONGKONG/205/1977 | Classical | 480 | **1920** | <10 | <10 |
| A/CHICKEN/HIDALGO/232/1994 | Classical | 800 | 1120 | 40 | 160 |
| A/EMU/NEWYORK/12716/1994 | Classical | 120 | 560 | <10 | <10 |
| A/MALLARD/NETHERLANDS/3/1999 | Classical | 680 | 1973 | **52** | 143 |
| A/AVIAN/NEWYORK/315883/2000 | Classical | 800 | 1600 | 40 | 280 |
| A/ENVIRONMENT/DELAWARE/1346/2001 | Classical | 1600 | 560 | 40 | 100 |
| A/MALLARD/SWEDEN/132/2002 | Classical | 800 | 1600 | 40 | 120 |
| A/MALLARD/SWEDEN/368/2002 | Classical | 1040 | 2160 | 60 | 200 |
| A/MALLARD/SWEDEN/718/2002 | Classical | 320 | 2240 | 35 | **100** |
|  |  |  |  |  |  |
| A/HONGKONG/156/1997 | Clade 0 | 1312 | 2427 | 120 | 230 |
|  |  |  |  |  |  |
| A/VIETNAM/1194/2004 | Clade 1 | <10 | 35 | <10 | <10 |
| A/INDONESIA/5/2005 | Clade 2.1 | <10 | <20 | <10 | <10 |
| A/SWAN/GERMANY/R651/2006 | Clade 2.2 | 20 | 80 | <20 | <20 |
| A/ANHUI/1RG/2005 | Clade 2.3 | * | 30 | <10 | <10 |

* refers to data that are unavailable
